# Mr. Hill's Case of Monstrosity

**Published:** 1810-07

**Authors:** John Wilkes Hill

**Affiliations:** Surgeon. Trinity Square


### *To the Editors of the Medical and Physical Journal.*

Gentlemen,

DEVIATIONS from the natural structure in the parts of man, do not lead to any immediate improvements in the practice of medicine, but they greatly tend to enlarge our knowledge of the animal economy, and may in this way be said to contribute to the advancement of the healing art.

The Case in question, which has lately occurred to me in my practice, and which I shall now attempt to describe, is not quite so complete in its kind as I could have wished, although very different in most respects from those numerous preparations I have lately had an opportunity of seeing in the Anatomical Museum of the Royal College of Surgeons.

On Monday, June 11, 1810, about ten in the morning, I was called to attend Mrs. ——, who was in the 7th month month of her pregnancy. She stated, that in coming down stairs with a large child in her arms, she had a sudden and very considerable discharge of water from the vagina, that she had not perceived any distinct motion of the child for two days. In consequence of the liquor amnii being thus discharged, I thought it right to ascertain, as near as possible the state of the os uteri, and accordingly prevailed on my patient to submit to an examination per vaginam. I observed the os uteri to be somewhat dilated, and very flaccid; the presence of the finger did not cause any uterine contraction whatever. I have attended the patient with four children before, and she was obviously larger in this pregnancy than in the preceding ones. As there was a total absence of pain, I conceived that it could be of no use to stop with her, and I consequently left her, previously giving directions to her nurse, to send for me as soon as her pains put on an appearance of regularity. Between seven and eight in the evening my attendance was again requested; the pains had now become frequent and regular, and on examination, the os uteri was considerably dilated, with a protrusion of portion of membrane, in a bag-like form; in a short time afterwards this membranous bag gave way, and another considerable quantity of water was evacuated; after this second discharge, the arm presented itself, and came down into the vagina, which appeared to me necessary to return; this I accomplished during the absence of the pains, and by bringing down at the same time the breech of the child, it was shortly expelled (in this position) into the world.

After its expulsion, on seeking for the funis, I could not find any regular appearance of cord; but in the place of its insertion into the abdominal parietes, there was a membranous bag, which in the birth of the child had been ruptured, that enveloped and enclosed all the viscera of the abdomen; on passing my finger to examine for the placenta, that mass was found detached, and of course it was soon brought away.

On a more accurate examination of this monstrosity, it was found that there was no muscular covering to the abdominal viscera, a total want of the common parietes, and in their place there was a membranous expansion, which appeared to be a continuation of peritonæum. This membranous expansion was adherent to the inner surface of the general membranes, surrounding the waters and child, yet formed a separate and complete cavity, in which were the stomach, liver, intestinal canal, spleen, and other viscera; the the funis was unusually short (in length about four inches), it run from the placenta, in the duplicature of the external membrane, to the liver, without forming at its entrance into the abdomen, any appearance of navel; the placenta was larger than common, but the funis much smaller; there was no appearance whatever of the external parts of generation in the usual place, but where the umbilicus is situated, there was a fissure, and in this was an appearance not unlike the labia externa separated, at the upper part of which was a small orifice, which proved to be the termination of the small intestines, opening into a smooth surface, where the caput coli is usually situated; in the centre was a rugous corrugated kind of membrane, which was considered by an eminent anatomist,* to bear a great similarity to the appearance of the inside of the bladder, with its anterior surface removed, as is occasionally observed in those monstrosities termed hermaphrodites. Internally, but not within the cavity of the pelvis, was the uterus, with its appendages, but there was no vagina or external opening from it; there was a considerable curvature of the spine, with the body bent towards the left side, and the femur on that side was much misplaced, in consequence of which, the left leg and thigh were turned upward and outward, and the left hip considerably higher than the right, so that at first sight there was a very great malformation of the pelvis; the chest, head, arms, and legs, were as perfectly formed to external appearance as those of a fœtus of seven months usully are.

I have the pleasure to say, the patient did well; but such was the prejudice of the family, that no intreaty or persuasion, on my part, could induce them to allow that strict examination of this important and interesting subject so necessary for public investigation.

I am, &c.

JOHN WILKES HILL, Surgeon.

*Trinity Square, June 22,* 1810.

---

* Mr. T. Blizzard.

CRITICAL

